# Community based needs assessment in an urban area; A participatory action research project

**DOI:** 10.1186/1471-2458-12-161

**Published:** 2012-03-07

**Authors:** Saeid Sadeghieh Ahari, Shahram Habibzadeh, Moharram Yousefi, Firouz Amani, Reza Abdi

**Affiliations:** 1 Department of Community Medicine, Ardabil University of Medical Sciences, University street, 5615746765, Ardabil, Iran; 2 Department of Internal Medicine, Ardabil University of Medical Sciences, University street, 5615746765, Ardabil, Iran; 3 Empowerment Institute, Bakeri street, 5624849658, Ardabil, Iran; 4 Department of ELT, Faculty of Humanities, University of Mohaghegh Ardabili, University Street, 5619911367, Ardabil, Iran

## Abstract

**Background:**

Community assessment is a core function of public health. In such assessments, a commitment to community participation and empowerment is at the heart of the WHO European Healthy Cities Network, reflecting its origins in health for all and the Ottawa Charter for Health Promotion. This study employs a participation and empowerment plan in order to conduct community assessment.

**Methods:**

The method of participatory action research (PAR) was used. The study was carried out in an area of high socio-economic deprivation in Ardabil, a city in the northwest of Iran, which is currently served by a branch of the Social Development Center (SDC). The steering committee of the project was formed by some university faculty members, health officials and delegates form Farhikhteh non-governmental organization and representatives from twelve blocks or districts of the community. Then, the representatives were trained and then conducted focus groups in their block. The focus group findings informed the development of the questionnaire. About six hundred households were surveyed and study questionnaires were completed either during face-to-face interviews by the research team (in case of illiteracy) or via self-completion. The primary question for the residents was: 'what is the most important health problem in your community? Each health problem identified by the community was weighted based on the frequency it was selected on the survey, and steering committee perception of the problem's seriousness, urgency, solvability, and financial load.

**Results:**

The main problems of the area appeared to be *the asphalt problem*, *lack of easy access to medical centers*, *addiction among relatives *and *unemployment of youth*. High participation rates of community members in the steering committee and survey suggest that the PAR approach was greatly appreciated by the community and that problems identified through this research truly reflect community opinion.

**Conclusions:**

Participatory action research is an effective method for community assessments. However, researchers must rigorously embrace principles of mutual cooperation, respect for public ideas, and a robust belief in community empowerment in order to pave the way for responsible and active citizen participation in the various stages of research.

## Background

Community-based participatory research (CBPR) has been identified as a key strategy for effectively reducing health disparities in underserved communities [1]. Assessing the health of a community through CBPR was identified as one of the core functions of public health in the Institute of Medicine's *The Future of Public Health *[2]. The Future of Public Health (1988) recommended that local public health agencies should "regularly and systematically collect, assemble, analyze, and make available information on the health of the community, including statistics on health status, community health needs, and epidemiologic and other studies of health problems [3]. However, even when assessments were completed, policy development and assurance mostly did not follow [4,5].

Strong historical roots of assessment can be found in England. John Graunt (1620-1674), an Englishman, is credited to be among the first demographers. His *Natural and Political Observations upon the Bills of Mortality *written in 1662 demonstrated that there was regularity in mortality and survivorship figures. Yet, William Farr, appointed the first "Compiler of Abstracts" at the General Register Office in July 1839, is generally said to be the first to make use of the standardized mortality rate to adjust for differences in age distribution in different subgroups [3].

### Community health assessment defined

Community health assessment should not be confused with clinical needs assessments, which are routinely performed during an initial visit to a medical care provider. Community health needs assessment produces information that is relevant to groups and is not focused on the medical needs of individuals so that treatment plans can be developed accordingly. Furthermore, community health needs assessment should not be confused with assessment of disease prevention services. Since health is not seen merely as the absence of disease, community health assessment, therefore, focuses on general well-being. Of course, in many cases, disease prevention and promotion of general health overlap [6].

Definitions of community health assessment (CHA) widely vary. While some definitions focus on data collection and analysis, others highlight the use of assessment data to develop objectives and action plans for health improvement [3]. A straightforward definition for CHA is "collecting and analyzing, and using data to educate and mobilize communications, develop priorities, garner resources, and plan actions to improve public health [7]. In this article, we use the term *Community Health Assessment *(CHA) to describe both the process and the product of assessment, in that population health data are essential to both CHA's process and products. We identify the major components of CHA as community engagement, data access, data analysis, and interpretation.

### Community participation and empowerment

Participation by local households would require optimal community engagement [8]. Assessment partnerships are encouraged in *Healthy People 2010 *and in state-level public health improvement plans such as *Healthiest Wisconsin 2010: A Partnership Plan to Improve the Health of the Public *[9]. A commitment to community participation and empowerment is at the heart of the *WHO European Healthy Cities Network *(WHOEHCN), reflecting its origins in health for all and the *Ottawa Charter for Health Promotion *[10]. Health promotion works through concrete and effective community action in setting priorities, making decisions, planning strategies and implementing them to achieve better health. At the heart of this process is the empowerment of communities, their ownership and control of their own endeavors and destinies [11]. The subsequent *Jakarta Declaration *(WHO, 1997) reinforces this focus, giving priority to increasing community capacity and empowering individuals. It emphasizes the necessity of participation, with actions being carried out by and with people, not on or to people [12].

Although rigorous evidence of the effectiveness of community participation in relation to health is limited, community participation is widely accepted to have many important benefits [13]. Key benefits include increasing democracy, mobilizing resources and energy, developing more holistic and integrated approaches, achieving better decisions and more effective services, ensuring the ownership and sustainability of programs, and empowering communities [14].

### Participatory action research

Participatory action research (PAR) is a research process that focuses on improving the quality of service by means of a self-reflecting process of exploring and solving problems [15,16]. The basic structure of PAR is an ever increasing spiral process of planning, acting, observing, reflecting, developing theory and re-planning [15]. Participation, collaboration and mutuality of all participants in all levels of research is effective in identifying and defining the problem, planning the research, collecting and interpreting the data, planning and evaluating the intervention and re-evaluating the problem in light of the new information generated from the implemented activities, and, finally, disseminating the information [17,18]. PAR works with a community, which is defined as a group of people who share a common interest and not necessarily a common geographical location. Empowerment and social change are important goals of PAR. Equality in sharing control and power are basic values of PAR. Through participation in the research process, disempowered participants are expected to lose their fear, and shame, gain self-confidence, self-esteem and control, and develop an understanding of their own value. PAR is highly relevant for work with oppressed and disempowered communities with self-help groups and for health education [16,19,20]. The researchers become essentially facilitators or catalysts, and participants become co-learners in PAR; nobody is considered the expert [20]. Insiders and outsiders work together as equals to solve problems. PAR is subjective and therefore not always neutral [17]. PAR involves commitment from all participants and requires mutual respect, trust, humility, adaptability and a holistic approach to problem solving. Listening, dialogue and negotiating consensus are strategies to achieve mutuality and empowerment. As stated previously, the PAR process is an open process that requires constant revisiting of previous levels with newly generated knowledge from actions taken, which then help to reshape the problem and resolve it at a deeper level [20].

This paper describes a local PAR project to conduct a community health assessment in an urban region of Ardabil, a city in the northwest of Iran. The primary goals of this study were to: 1) demonstrate how health related needs could be assessed through a PAR approach to community participation in an urban community inside a developing country; and 2) encourage community groups and non-state organizations to collaborate to conduct health-related research. The broadness of the issue and diversity of community groups, made both goals challenging from the start.

## Methods

### Study design and community selection

A community PAR was conducted drawing on theories of community mobilization, participation, and empowerment. The steps included 1) establishing the Steering Committee 2) deciding on methods 3) identifying trusted and interested people to form Executive Committees 4) transferring knowledge 5) collecting and weighting data and 6) interpreting data and prioritizing needs.

A local requirement that stipulates that any community based program should be based on a formal demand by the community made us choose a potentially demanding area, based on the criteria of 'low socio-economic status', 'an abundance of various health problems', and a persistent demand on the part of the residents for improvement. The existence of a non-state health center and a high probability of participation were other criteria for choosing this location. At a meeting with delegates from Health Department of Ardabil Medical University, Mayoralty, and Welfare Organization, an area of about 20000 inhabitants was selected for the study site.

Our research project followed a set of prior activities that were undertaken by some members of the current project with the aim of establishing relationships with the local people and winning their trust. The earlier activities included identifying the trusted individuals, those with philanthropic interests, and those who were interested in local development and trust-building projects. Earlier projects involved repairing small open sewer canals, lighting pathways, holding leisure time classes, building sport teams, allocating library space inside the non-state health center of the region, and providing consultation services. All of the above services were made possible through cooperation between the community representatives and non-state organization agents, who managed to involve and attract the attention of the highest authority of the province in the process.

These successful experiences paved the way for this study. The research committee examined the profiles of the trusted and interested people in voluntary philanthropic activities and outlined the study procedures. Twelve Executive Committees were formed by representatives from 12 Blocks that were selected after considering physical texture and pathways following the blocking system of local community development center.

The most important challenge of this study was to encourage academic researchers and officials of health system to believe in the fact that people can participate in health domain research and be empowered to help conduct health research more effectively.

### Involving the community development center and selecting executives

In order to encourage the Community Development Center (CDC) of Ardabil to participate in this study, the general outline was discussed with CDC officials, agents from *Farhikhteh *non-state organization, and local people, during three 2-hour face to face meetings. Finally, the Executive Team of research project was decided mostly from among the local people and a few number of university colleagues. An attempt was made to select the majority of Executive Team members of the study from among the non-state organizations and local people. The ratio of the university colleagues to other members was 1 to 7. The members of the instruction, documentation, supervision, coordination, interview, and enquiry teams were selected from among the community members of CDC and the Farhikhteh institute. Rigorous care was taken to limit the role of the academic members to instruction and other technical aspects and much of the research task were delegated to the community groups in spite of numerous difficulties.

### Knowledge transfer and empowerment

The different methods of community assessment were presented through lectures to all members of the Steering Committee. The group preferred the 'focus group' technique to the other presented methods. The members of the project Executive Team and the representatives of the twelve blocks, who were selected from among interested people based on the documents of the *Social Research Center*, attended focus group workshops for two months. In addition, a questionnaire designing workshop and the data entering methods were hold for the community members of the project. The instruction prepared members for full participation; in practice, much of the job was delegated to ordinary members of the Executive Committees.

### Method of data collection

The trained community agents of the Executive Committees held group discussions in the twelve blocks with an average attendance of 8 to 14 neighborhood residents with the retention rate of about 70%. On the whole, three group discussions were held in every block by agents who were fluent in both Turkish and Persian.^1 ^The invited people included local retailers, state employees, housewives, pensioners, trustees, and active youth from the local blocks. The people attending the discussions were also supposed to act as facilitators of the research and prepare the community for full participation.

A note-taker recorded the details of every discussion. The workshops took place in April through May, 2006. The venues for the workshops were decided based on the convenience of each individual group and included the neighbor's homes, local mosques or CDC rooms. During the workshops, the purpose and process of the research was thoroughly explained.

The research process was started with the following statement: "*what is the most important problem in your community's health?"*. The agents were asked to tell people that "As a member of our community, we want to understand the problems better. It is necessary to know the answer to this question according to your priorities, so that we can suggest an appropriate intervention to health and other officials, and then implement the intervention, and assess the results of our efforts."

From the beginning, it was made clear to the community that health system officials and relevant domains were expected to allocate considerable amounts of time and money on an annual basis to improve health condition. However, the main challenge was to decide on the priorities from the perspectives of the locals.

Each block team was given the mission to discover *the most important problems *in their community.

After finishing the workshops, the results were reported to the Steering Committee by the representatives of the groups. The final procedure was agreed to by the Steering Committee with the cooperation of agents of *Farhikhteh Institute *and representatives of twelve local areas.

Subsequently, in order to assess the needs from the perspectives of the households of the blocks, the Steering Committee planned more workshops to empower the community groups to design the questionnaire and conduct interviews. Three 1-day workshops were planned and implemented in July through August, 2006.

The Steering Committee, representatives of twelve blocks and *Farhikhteh *institute agreed on a questionnaire which included 60 yes-no items. The items were related to the general problems of local people such as health, security, economy, employment, and education. Subsequently, a final orientation session was held for all the local interviewers to practice completing the questionnaire.

The community interviewers of 12 local areas and their supervisors, from among the members of the Executive Committees interviewed 30 households from the 12 blocks and repeated it after a 14-day interval in order to check the reliability of the instrument, which was found to be 0.76.

Six hundred households were interviewed in September 2006. The target households were selected through cluster random sampling using the CDC database. Considering the population (20,000) and the average number of family members (4.3) in Iran [20], 600 households equaled about 15% of the households. It should be noted that the demographic information of the participants was not systematically gathered. The supervisors examined the daily delivered questionnaires and randomly checked some households for quality assurance purposes.

### Method of data analysis

During the Steering Committee's meetings, the necessity of including diverse groups of people was discussed. The best method of implementing community assessment was also discussed. Finally, the Steering Committee decided to apply a mixed model containing surveys and focused group discussion in the local areas.

The first set of data was produced following analysis of the priorities offered by 12 local groups which represented each block. Then, face-to-face interviews were carried out with [almost all] 600 households of the selected area, to create a second dataset. With consistent supervision and training, the community groups entered the data into the computer as planned. They cooperated with a statistician to analyze the data. Finally, the output of the data which comprised five main problems from the perspective of 600 households was produced.

### Ethical considerations

This study was approved in the research committee of Ardabil University of Medical sciences, which considers and verifies the research proposals both academically and ethically. It should also be noted that participation in this project has been voluntary for all the community representatives and the agents of Farhikhteh institute of Ardabil. In the first meeting, their option to leave or continue the study was explained to them formally at the beginning and during the study. The researcher after acknowledging their participation in the project ensured the privacy of the data. Additionally, an attempt was made to employ both female and male colleagues to observe the religious and cultural norms and values.

## Results

In the first stage of analysis, the needs of 12 local areas were identified. The number of identified needs for the neighborhoods varied from 8 to 24. As it can be seen in Table 1, the five prioritized problems for each neighborhood are related but not limited to the health domain.

**Table 1:** The leading priorities of local areas

Blocks	Priority 1	Priority 2	Priority 3	Priority 4	Priority 5
1	Littered neighborhood	The asphalt problem	Open sewer	Poor or no Snowplowing in the winter	The problem of neighborhood with iron sellers market
2	The asphalt problem	Littered neighborhood	Inefficient rubbish collection system	Darkness of the neighborhood at night	Open sewer
3	The asphalt problem	Inefficient rubbish collection system	The problem of water canals	Open sewer	Lack of closed sewer system
4	Poor or no Snowplowing in the winter	Inefficient rubbish collection system	The asphalt problem	Water shortage	Absence of house plaques and alley names
5	The asphalt problem	Water shortage	Inefficient rubbish collection system	Electrical current Instability	Lack of public transportation vehicles
6	The asphalt problem	High unemployment rate	Inefficient rubbish collection system	Open sewer	Littered neighborhood
7	The asphalt problem	High unemployment rate	inefficient rubbish collection	Open sewer	Littered neighborhood
8	The asphalt problem	High unemployment rate	Littered neighborhood	Darkness of neighborhood at night	The stench of streams
9	littered neighborhood	Water shortage	Lack of welfare, education and recreational facilities	Littered neighborhood	High unemployment rate
10	lack of welfare, education and recreational facilities	Lack of public transportation vehicles	Defective slope of water canals	Lack of access to mosque and Basij base	The asphalt problem
11	The asphalt problem	The problem Of water canals	Financial problems and destitution of people	High unemployment rate	Sanitary/health problems of the region
12	Inefficient rubbish collection system	High unemployment rate	Darkness of neighborhood at night	The housing problem	Poor or no Snowplowing in the winter

In Table 2, the results of the analysis of frequency of the problems, from the point of view of 600 households, are displayed.

**Table 2:** The rank of problems according to the frequency from the viewpoint of people (calculated as the average number of people in any 100 people who mentioned the problem)

Rank	The problem	Frequency
1	lack of easy access to health and medical center	97
2	Existence of mechanical repair shop in the local area	96.4
3	lack of mosques or little people's activity in the mosques	87
4	Lack of parking lots	83.3
5	Lack of interest-free loan fund in the local area	83
6	Lack of fruit/vegetable market and department stores	78
7	Inappropriate rubbish disposal	67.8
8	Poor provision of subsidized milk	67.7
9	littered neighborhood	66.8
10	The asphalt problem	66.6
11	Absence or shortage of sport facilities	65
12	The problem of pathways lightings	62.7
13	Absence or remoteness of feminine high school	61.8
14	The shortage of public transportation vehicles	54.8
15	Disturbance of hooligan youth in the local area	54.4
16	Unemployment of youth	45.5
17	The problem of old water pipes and water shortage in the local area	34.2
18	Insecurity when leaving homes	32.4
19	Addiction among relatives	30.4
20	Low economic welfare indices	29
21	Enormous cost of children's high education	25.4
22	The housing problem	21.5

In the next stage of research, the Steering Committee decided on some more criteria to produce more practical results. The four criteria that were agreed on were: seriousness, urgency, solvability, and financial burden of the problems, which received weights (quotients) of 8.6, 7.5, 5.5, and 4.8, respectively. The frequency criterion received a weight of 6.8. To arrive at these weights, all 30 members of the Steering Committee assigned a weight score of 1-10 to the above five criteria and then the results were averaged out.

When the five criteria and relevant weights were decided, the Committee met again and all 30 members provided a value number of 1-100 to each problem (e.g. lack of adequate pathway lighting) in terms of its seriousness, urgency, solvability, and financial burden. Then, the five values were multiplied by the relevant weight to yield the final score for each problem which appears in Table 3.

**Table 3:** Final prioritization of the problems of urban area under study

Rank	The problem	Total score
1	The asphalt problem	2508.3
2	Lack of easy access to health and medical centers	2317.2
3	Addiction among relatives	2315
4	Unemployment of youth	2285.8
5	Inappropriate rubbish disposal	2248.7
6	Absence and remoteness of feminine high school	2128.1
7	The problem of pathways lightings	2102.4
8	Absence or shortage of sport facilities	2004.2
9	Existence of mechanical repair shop in the local area	1984.2
10	Lack of parking lots	1914.2
11	The shortage of public transportation vehicles	1894.9
12	Lack of fruit/vegetable market and department stores	1883.4
13	littered neighborhood	1859.7
14	Low economic welfare indices	1770.5
15	lack of interest-free loan fund in the local area	1765.1
16	Poor provision of subsidized milk	1653
17	Enormous cost of children's high education	1632.3
18	Disturbance of hooligan youth in the region	1624.3
19	Lack of mosques or their low level of activity	1501.3
20	The problem of old water pipes and water shortage in the local area	1485.8
21	Insecurity when leaving the homes	1453.8
22	The housing problem	1097.5

## Discussion

This participatory action research demonstrated that the availability of trusted and philanthropic people could be very helpful at the beginning of the project. This study also revealed that when assessment of the health problems of a community is carried out, other social problems may be observed that influence the community's general health. As confirmed by the data in Table 3, health is influenced by an array of social factors [21,22].

Working "with people and for people" during the project indicated that efforts for establishing relationships, empowerment, trusting key roles to people, and involving them in health research can pave the way for high community participation. However, convincing people to trust and join the project was a real challenge at the beginning, which was resolved by the perseverance and negotiation of the certain members of the Steering Committee with the trusted group.

The results of study clarified that in working with the community, researchers should ignore their presuppositions, and let participants discover their own problems and needs, which is a crucial step in empowerment.

Participatory researchers in developing countries such as Iran allocate most of their energy to coping with local rules, getting the approval of participatory research projects, and facing objections from traditional researchers.

This study demonstrated that active community participation can be achieved if the following conditions are met:

1. Acknowledging the key role of people in designing and actually conducting studies;

2. Providing adequate training in research methods;

3. Building trust and empowerment;

4. Seriously taking the community's viewpoint into account;

5. Crating a sense of responsibility in the community;

6. Involving a non-state organization in the research as a bridge between the community and the state; and

7. Communicating research results with participants in public forums and newspaper articles.

However, this study could have been more useful if the following limitations were not present. In the first place, we could not secure a full participation of authorities from non-health departments. Secondly, the demographic details was not gathered which could have enriched the interpretation of the data. Thirdly, we could not attract a proportionate participation of women due to cultural constraints. Finally, our project was the first in type in the region both for the members of the steering committee and also the general participants, which frequently resulted in slowing the procedure.

## Conclusions

PAR is very applicable for community assessment. However, researchers must rigorously take into account the caveats of mutual cooperation, respect for public ideas, and a robust belief in community empowerment in order to pave the way for people to feel responsible and actively take part in the various stages of research.

## Endnotes

The native language of people in Ardabil Province is Turkish, while the official language is Persian. During late decades, the local people have used Turkish for oral conversation and Persian for written communication. Only a quite small number of people are able to read and write in Turkish. However, in accordance with the current traditions and convenience of region, the group discussions were performed in Turkish, but recorded in Persian.

## Abbreviations

SDC: Social Development Center; CBPR: Community-Based Participatory Research; CHA: Community Health Assessment; IOM: Institute of Medicine; WHOEHCN: WHO European Healthy Cities Network; PAR: Participatory action research.

## Competing interests

The authors declare that they have no competing interests.

## Authors' contributions

All authors made a substantial contribution to and the design and implementation of the study and were involved in drafting and reviewing the manuscript. All authors have read and approved the final manuscript.

## Pre-publication history

The pre-publication history for this paper can be accessed here:

http://www.biomedcentral.com/1471-2458/12/161/prepub
